# The impact of sarcopenia on low back pain and quality of life in patients with osteoporosis

**DOI:** 10.1186/s12891-022-05086-2

**Published:** 2022-02-11

**Authors:** Shoji Iwahashi, Ryuki Hashida, Hiroo Matsuse, Eriko Higashi, Masafumi Bekki, Sohei Iwanaga, Koji Hara, Takahiko Higuchi, Yohei Hirakawa, Asami Kubota, Hiromi Imagawa, Yoko Muta, Kazuhito Minamitani, Tatsuhiro Yoshida, Kimiaki Yokosuka, Kei Yamada, Kimiaki Sato, Naoto Shiba

**Affiliations:** 1 Department of Orthopedics, Kurume University School of Medicine, 830-0011 Kurume, Japan; 2 Division of Rehabilitation, Kurume University Hospital, 67 Asahi-machi, 830-0011 Kurume, Fukuoka Japan; 3 Munakata Suikokai General Hospital, 5-7-1 Himakino, 811-3298 Fukutsu, Japan; 4 Department of Orthopaedics, Munakata Suikokai General Hospital, 5-7-1 Himakino, 811-3298 Fukutsu, Japan

**Keywords:** Osteoporosis, Adults spinal deformity, Sarcopenia, Quality of life, Sagittal alignments

## Abstract

**Purpose:**

Osteoporosis combined with sarcopenia contributes to a high risk of falling, fracture, and even mortality. However, sarcopenia’s impact on low back pain and quality of life (QOL) in patients with osteoporosis is still unknown. The purpose of this study is to investigate low back pain and QOL in osteoporosis patients with sarcopenia.

**Methods:**

We assessed 100 ambulatory patients who came to our hospital for osteoporosis treatment. Low back pain was evaluated using the Visual Analogue Scale (VAS) with 100 being an extreme amount of pain and 0 no pain. The Japanese Orthopaedic Association Back Pain Evaluation Questionnaire (JOABPEQ) score was used to assess QOL after adjustment for age, history of vertebral fracture, and adult spinal deformity. Differences in low back pain intensity assessed by VAS between groups were evaluated by the Willcoxon rank-sum test. Covariance analysis was used to assess QOL. All data are expressed as either median, interquartile range, or average, standard error.

**Results:**

Patients were classified into the sarcopenia group (*n* = 32) and the non-sarcopenia group (*n* = 68). Low back pain intensity assessed by VAS was significantly higher in the sarcopenia group than in the non-sarcopenia group (33.0 [0-46.6] vs. 8.5 [0-40.0]; *p* < 0.05). The subscales of the JOABPEQ for low back pain were significantly lower in the sarcopenia group than in the non-sarcopenia group (65.0 ± 4.63 vs. 84.0 ± 3.1; *p* < 0.05).

**Conclusion:**

In this cross-sectional study, sarcopenia affected low back pain and QOL in ambulatory patients with osteoporosis. Sarcopenia may exacerbate low back pain and QOL.

## Introduction

Muscle mass has been observed to decrease with age at a rate of approximately 1% annually after age 40 [1]. Sarcopenia is defined as the loss of skeletal muscle mass and strength that occurs with advancing age [2]. Osteoporosis is defined as a skeletal disorder characterized by compromised bone strength leading to an increased risk of fracture [3]. Both sarcopenia and osteoporosis are geriatric diseases that decrease activity in daily living, and are interconnected physically and chemically. Recently, combined osteoporosis and sarcopenia are called “osteosarcopenia” [4]. Osteosarcopenia contributes to a high risk of falling, fracture, and even mortality [5]. Since osteosarcopenia is a new concept, studies of osteosarcopenia are insufficient. Thus, it remains unclear how sarcopenia affects low back pain and quality of life (QOL) in patients with osteoporosis.

Aging and decline of physical activity lead to decreased QOL. Spinal misalignment induced by loss of skeletal muscle mass and vertebral fracture also leads to decreased QOL [6, 7]. In particular, increased thoracic kyphosis leads to decreased QOL in patients with osteoporosis [8]. The SRS-Schwab classification system is an assessment scale for spinal deformity using radiographic parameters including the sagittal vertical axis, pelvic tilt (PT), and pelvic incidence minus lumbar lordosis (PI-LL) [9]. Among them, PT is one of the most important parameters for assessing spinal deformity, PT is significantly correlated with pain, disability, and QOL [10].

In this cross-sectional study, we hypothesized that osteoporosis patients with sarcopenia would have more low back pain and poorer QOL than osteoporosis patients without sarcopenia. The purpose of this study was to investigate the effect of sarcopenia on low back pain and QOL in ambulatory patients with osteoporosis.

## Methods

### Ethics

The study protocol conformed to the ethics guidelines of the Declaration of Helsinki, as reflected in prior approval given by the institutional review board of Munakata Suikokai General Hospital (approval ID: 19,002). Informed consent from patients was obtained using an opt-out approach.

### Patients

We did a cross-sectional study and assessed 100 ambulatory patients when they came to our hospital (Munakata Suikoukai General Hospital, Japan) for osteoporosis treatment between January 2018 and December 2018. We summarized inclusion and exclusion criteria in Table 1. According to the Japanese Society for Bone and Mineral Research, we diagnosed osteoporosis and osteopenia [11].

**Table 1:** Inclusion and exclusion criteria

Inclusion Criteria	Exclusion criteria
Patients 60 years of age or over, with osteoporosis or osteopenia Osteoporosis: History of fragility fracture and bone mineral density (BMD) <80% of the young adult mean, or BMD<70% Osteopenia: BMD was 70% to 80% of the young adult mean	Unable to stand Unable to walk 10 meters Unable to be measured in various physical tests Pacemaker Long-term steroid use Fresh vertebral fracture Hemiplegia

### Diagnosis of sarcopenia

We assessed sarcopenia according to the Asian Working Group for Sarcopenia guidelines [12]. (1) Cutoff values for handgrip strength are <26 kg for men and <18 kg for women. The cutoff value for walking speed is the usual gait speed of <0.8 m/s. Patients with either grip strength or walking speed less than the cutoff value are suspected to have sarcopenia. (2) In cases of suspected sarcopenia, the next-step is to evaluate muscle mass. Cutoff values for skeletal muscle mass index (SMI) are 7.0 kg/m2 for men and 5.7 kg/m2 for women measured by bioimpedance analysis. (3) Patients with less than the muscle mass cutoff are diagnosed with sarcopenia.

### Outcome measures

#### Bone mineral density

Areal BMD of the posterior-anterior lumbar spine was measured by dual-energy x-ray absorptiometry using a Hologic QDR 4500 A densitometer (Hologic, Waltham, MA). All scans of an individual subject were performed using the same densitometer. Quality control measurements were performed daily with a Hologic anthropomorphic spine phantom.

### Isometric knee extension strength

Isometric knee extension strength on the dominant side was measured at discharge using a manual muscle strength monitor (Mobie; Sakai Medical Co., Ltd.) [13]. Patients sat on the edge of a bed with their feet not touching the floor and with their arms crossed in front of their body. The highest value of three assessments was used in the analysis.

### Evaluation of pain

Low back pain, pain from the buttocks to lower limbs, and numbness of the buttocks to lower limbs were evaluated using a Visual Analogue Scale (VAS) with 100 being an extreme amount of pain and 0 no pain.

### Spinal alignment

**Fig. 1:**
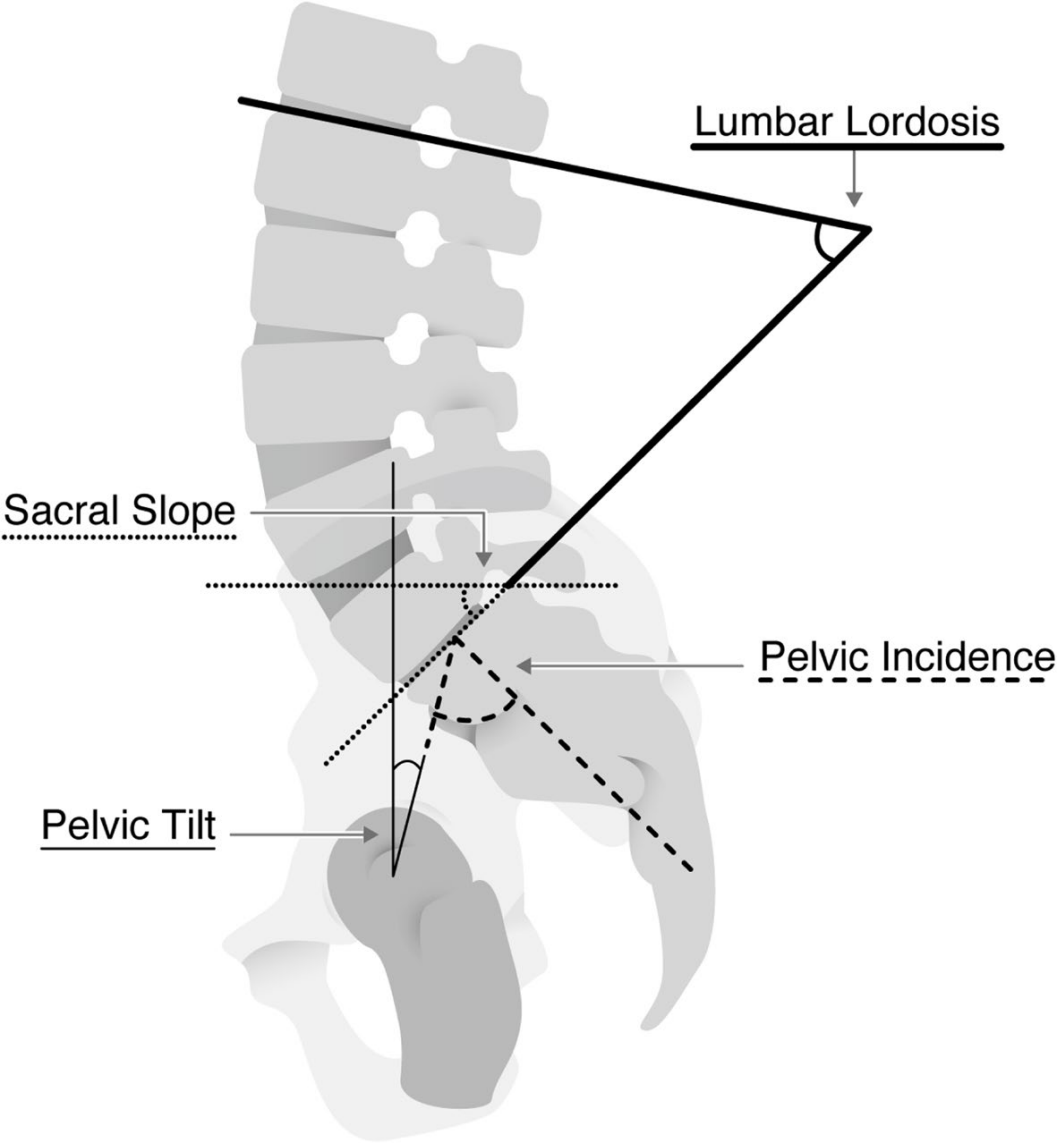
Radiographic parameter of spinal alignment. LL is measured from the inferior endplate of T12 to the superior endplate of S1. SS is measured as the angle between the sacral plate and the horizontal line.PT is measured by the angle between the vertical and the line through the midpoint of the sacral plate to the femoral heads axis. PI is measured as the angle between the line perpendicular to the sacral plate at its midpoint and the line connecting this point to the femoral head’s axis. “PI = PT -SS. Abbreviations: LL, lumbar lordosis; PI, pelvic incidence; SS, sacral slope; PT, pelvic tilt

The patient was radiographed at the clavicle position [Poosiripinyo T (2017) J Orthop Sci 22 (1):34-37.]. LL, PI, PT, and sacral slope (SS) were measured from various lumbar spine lateral views (Fig. 1). We defined adult spinal deformity as PT>30 degrees, based on the SRS-Schwab classification [10].

### Disease-specific QOL measure for patients with low back pain

The Japanese Orthopaedic Association Back Pain Evaluation Questionnaire (JOABPEQ) was designed for the assessment of disease-specific QOL and low back pain and lumbar spinal disease. It is a disease-specific tool that contains 25 items tapping into five subscales: low back pain (four items), lumbar function (six items), walking ability (five items), social function (four items), and mental health (seven items). The score for each subscale ranges from 0 to 100, with higher scores indicating better conditions. The JOABPEQ is used in Japan and other countries, and its reliability has been proven [14]. Recently, the JOABPEQ is used to assess low back pain patients and can be used to assess other lumbar spine disorders such as lumbar canal stenosis, lumbar disc herniation, and neuropathic pain in a low back pain patient [14].

### Statistical analysis

All the statistical analysis were performed by JMP Version 14.0 statistical software (SAS Institute Inc., Cary, NC, USA), with *P* < 0.05 considered statistically significant in all cases. Differences in patient characteristics at the time of visit and JOABPEQ results between the sarcopenia and non-sarcopenia groups were evaluated by the Willcoxon rank-sum test. QOL is affected by age, history of vertebral fracture and adult spinal deformity, so we used analysis of covariance to adjust these factors. Pearson correlation coefficient was used to determine the correlation between SMI and spinal alignment parameters or the JOABPEQ. The Pearson correlation coefficient measures the strength of the linear relationship [15, 16]. It ranges from -1 to 1. The correlation coefficient value may be expressed from very weak to very strong in increments of 0.2. 0 to 0.19 is considered very weak, either positive or negative, and 0.8 to 1.0 is considered very strong. A multivariate Cox regression analysis with stepwise variable selection was used to identify independent variables associated with low back pain in the JOABPEQ. All data are expressed as either median, (interquartile range [IQR]), range, or average, standard error.

## Results

### Patient characteristics at the time of visit

Patients were classified into sarcopenia (*n* = 32) and non-sarcopenia groups (*n* = 68), and their clinical characteristics at the time of visit are shown in Table 2. Patients in the sarcopenia group were significantly older than those in the non-sarcopenia group (*p* < 0.05). Body mass index (BMI) and SMI were significantly lower in the sarcopenia group than in the non-sarcopenia group(*p* < 0.05). The ratio of history of vertebral fracture and adult spinal deformity was significantly higher in the sarcopenia group than in the non-sarcopenia group (*p* < 0.05).

**Table 2:** Patient characteristics at the time of visit

	Sarcopenia (*N* = 32)		Non-sarcopenia (*N* = 68)		p
	Median (IQR)	Range (min-max)	Median (IQR)	Range (min-max)	
Age	81.5 (74.5-87.8)	63-97	71.5 (68.3-80.0)	60-91	0.0001
Sex (female/male)	28/4		60/8		0.9159
BMI (kg/m^2^)	20.7 (18.8-23.1)	14.3-26.3	22.5 (20.7-24.7)	17.5-32.2	0.0072
SMI (kg/m^2^)	5.1 (4.7-5.5)	4.2-6.8	6.1 (5.7-6.7)	4.9-7.9	<.0001
BMD (femoral neck)	0.50 (0.42-0.56)	0.38-0.83	0.56 (0.50-0.64)	038-0.90	0.0079
Knee extension torque (kgf/kg)	11.7 (6.4-15.8)	3.4-20.3	17.1 (13.1-21.5)	3.9-46.3	<.0001
History of vertebral fracture (N)	19(55.88%)		14(20.89%)		0.0004
Adult spinal deformity (N)	14(43.75%)		12(17.91%)		0.0063
Pelvic Tilt (°)	29.0 (21.2-37.8)	8.7-57.0	22.0 (16.0-28.0)	4.0-39.7	0.005
Lumbar Lordosis (°)	36.0 (26.3-49.8)	12.0-68.0	43.0 (36.0-52.9)	3.0-79.0	0.0445
Pelvic Incidence (°)	54.0 (49.0-68.5)	38.0-91.0	55.0 (50.0-61.0)	34.0-85.0	0.6614
Sacral Slope (°)	28.0 (23.3-36.8)	13.0-56.2	33.2 (27.0-40.0)	2.0-54.0	0.0441

*Abbreviations: BMI* body mass index, *BMD* bone mineral density, *SMI* skeletal muscle mass, *VAS* Visual Analogue Scale

### Bone mineral density, Isometric knee extension strength and Evaluation of Pain

BMD, and isometric knee extension torque were significantly lower in the sarcopenia group than in the non-sarcopenia group ((BMD (g/cm^2^):0.50 [0.42-0.56] vs. 0.56 [0.50-0.64]; *p* = 0.0079, isometric knee extension strength (kgf/kg): 11.7 [6.4-15.8] vs. 17.1 [13.1-21.5]; *p* < 0.0001)). Low back pain intensity assessed by VAS was significantly higher in the sarcopenia group than in the non-sarcopenia group(33.0 [0-46.6] vs. 8.5 [0-40.0]; 0.0312). There was no significant difference in VAS for pain from buttocks to lower limbs and numbness from buttocks to lower limbs(Pain from buttocks to lower limbs assessed by VAS (mm):0 [0-0] vs. 0 [0-3.0]; 0.5409, Numbness of buttocks to lower limbs assessed by VAS (mm): 0 [0-0] vs. 0 [0-0]; *p* = 0.8124).

### Spinal Alignment

PT in the sarcopenia group was significantly higher than in the non-sarcopenia group (*p* < 0.05). LL and SS were significantly lower in the sarcopenia group than in the non-sarcopenia group (*p* < 0.05). There was no significant difference in PI (Table 2).

### JOABPEQ (Disease-specific QOL) before adjusted

The results for QOL evaluated with the JOABPEQ are shown in Table 3. The subscales for low back pain, lumbar function, walking ability, and social life function were significantly lower in the sarcopenia group than in the non-sarcopenia group (*p* < 0.05). No significant difference in mental health was found between the two groups.

**Table 3:** Japanese Orthopaedic Association Back Pain Evaluation Questionnaire (JOABPEQ: Disease-specific QOL) before adjusting

	Sarcopenia (*N* = 32)		Non-sarcopenia (*N* = 68)		p
	Median (IQR)	Range (min-max)	Median (IQR)	Range (min-max)	
Low back pain	50 (43-100)	14-100	100 (71-100)	29-100	0.0003
Lumbar function	79 (17-83)	0-100	83 (75-100)	8-100	0.0031
Walking ability	64 (29-91)	0-100	93 (66-100)	0-100	<.0001
Social life function	65 (30-78)	0-100	78 (57-100)	14-100	0.0014
Mental health	54 (49-74)	21-94	63 (51-78)	39-96	0.1041

### JOABPEQ (Disease-specific QOL) after adjusted by age, history of vertebral fracture, and adult spinal deformity

The subscale score for low back pain was significantly lower in the sarcopenia group than in the non-sarcopenia group (*p* < 0.05). No significant difference between the two groups was found for lumbar function, walking ability, social life function, and mental health (Table 4).

**Table 4:** Japanese Orthopaedic Association Back Pain Evaluation Questionnaire (JOABPEQ :Disease-specific QOL) adjusted by age, history of vertebral fracture, and adult spinal deformity

	Sarcopenia (*N* = 32)	Non-sarcopenia (*N* = 68)	p
Low back pain	64.44 ± 4.42	84.25 ± 3.10	0.0005
Lumbar function	62.98 ± 4.84	73.31 ± 4.07	0.0995
Walking ability	67.55 ± 4.78	75.26 ± 3.42	0.17777
Social life function	63.08 ± 4.19	71.22 ± 3.53	0.13298
Mental health	61.94 ± 3.04	65.66 ± 2.18	0.3057

Data are given as means and standard error

### Correlation between variables

**Fig. 2:**
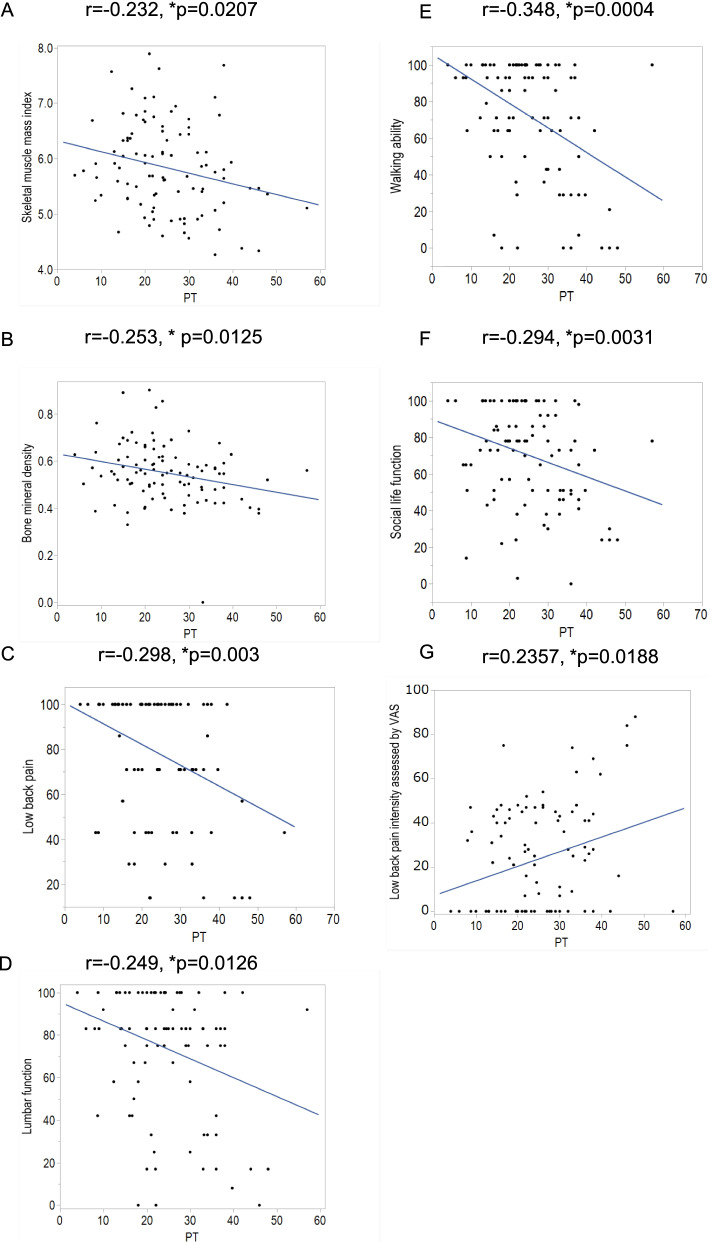
Correlations with pelvic tilt (PT). A statistically significant negative correlation was observed between PT and both skeletal muscle mass (**A**) and bone mineral density (**B**). A statistically significant negative correlation was observed between PT and the subscale scores of JOABPEQ for low back pain (**C**), lumbar function (**D**), walking ability (**E**), and social life function (**F**). A statistically significant positive correlation was observed between PT and Low back pain intensity assessed by VAS (**G**). Abbreviations: JOABPEQ, Japanese Orthopaedic Association Back Pain Evaluation Questionnaire; PT, pelvic tilt, VAS; Visual Analogue Scale

Correlations done for spinal alignment revealed a significant negative correlation between PT and both SMI and BMD (SMI; *r* = -0.232; *p* = 0.0207, BMD; *r* = -0.253; *p *= 0.0125). PT had a significant negative correlation with all categories in the JOABPEQ except for mental health (low back pain; *r* = -0.298; *p* = 0.003, lumbar function; *r* = -0.249; *p* = 0.0126, walking ability; *r* = -0.348; *p* = 0.0004, social function; *r* = -0.294, *p* = 0.0031, mental health; *r* = -0.0436, *p* = 0.6684). PT had a significant positive correlation with low back pain intensity assessed by VAS (*r* = 0.2357; *p *= 0.0188) (Fig. 2).

### Independent factors associated with low back pain in JOABPEQ

A multivariate Cox regression analysis was done with variables related to low back pain; age, BMI, gender, PT, LL, SS, BMD, SMI, gait-speed, grip, and sarcopenia. Sarcopenia and walking speed were the only significant factors for low back pain in the JOABPEQ (sarcopenia: estimate -9.694; 95% confidence interval -15.378 — -4.011; *p* = 0.010, walking speed: estimate 15.186; 95% confidence interval 3.027 — 27.345; *p* = 0.0149).

## Discussion

We assessed QOL and low back pain in ambulatory patients with osteoporosis. The ratio of sarcopenia was 32% in this study. Low back pain intensity assessed by VAS was significantly higher in the sarcopenia group than in the non-sarcopenia group. A multivariate Cox regression analysis extracted sarcopenia as the independent factor associated with back pain in the JOABPEQ. Sarcopenia might affect QOL and low back pain in patients with osteoporosis.

### Ratio of sarcopenia

In this study, we investigated the ratio of sarcopenia in osteoporosis patients. The proportion of osteoporosis patients with sarcopenia was 32% (average age 76.4 years). Yoshimura et al. investigated 1,099 Japanese patients with osteoporosis (average age 72.1 years) and reported a sarcopenia ratio of 19.1% [17]. They also showed a yearly increase with age of about 2.0% (male 2.2%, female 1.9%) in the ratio of sarcopenia in patients with osteoporosis. Morley et al. reported that the ratio of sarcopenia varies by age, with the ratio in a 60- to 70-year-old group of 5-13% and a ratio of 11-50% for those over 80 [12]. Taken together, the higher average age of our patients may have influenced the ratio of sarcopenia.

### Low back pain and sarcopenia

Low back pain intensity assessed by VAS in the sarcopenia group was significantly higher than in the non-sarcopenia group in this study. Sarcopenia was a significant factor for low back pain in JOABPEQ using multivariate Cox regression analysis. Sakai et al. investigated the relationship between low back pain and sarcopenia. The ratio of sarcopenia was 40.0% in the low back pain group and 26.6% in the non-low back pain group. Low back pain is correlated with SMI rather than with BMD. They concluded that sarcopenia was associated with low back pain [15]. Moreover, Spinal alignment including PT is one of the causes of low back pain. Schwab found PT is significantly correlated with an Oswestry Disability Index Score which suggests low back pain (cut off of PT is 22) [10]. Eguchi et al. reported that low back pain had a negative correlation with SMI and a positive correlation with PT, suggesting that sarcopenia may be associated with low back pain as a result of posterior pelvic tilt [16]. PT was significantly higher in the sarcopenia group than the non-sarcopenia group in this study (29.0 vs.22.0). PT was also significantly correlated with low back pain intensity assessed by VAS. Thus, our results were similar to the previous reports. Sarcopenia may be related to posterior pelvic tilt and low back pain.

### QOL and sarcopenia

Sarcopenia affects not only physical function but also fracture and mortality, although it was previously unclear that sarcopenia affects QOL in patients with osteoporosis. We first showed that sarcopenia affects QOL in patients with osteoporosis in this study. We assessed QOL using the JOABPEQ in this study. Subscale scores for low back pain, lumbar function, walking ability, and social life function were significantly lower in the sarcopenia group than in the non-sarcopenia group. In particular, the subscale score for low back pain was significantly lower in the sarcopenia group than in the non-sarcopenia group after adjusting for age, history of vertebral fracture, and adult spinal deformity. We also investigated the relationship between spinal alignment and both SMI and QOL by using the Pearson correlation coefficient. PT was significantly correlated with each item except for mental health in the QOL assessment by JOABPEQ and also significantly correlated with SMI. Appendicular and trunk muscle mass form the pelvic/lumbar stabilization structure. Eguchi et al. reported that sarcopenia was one of the causes for spinal deformity affecting QOL. They showed that PT is an important factor that affects QOL in patients with scoliosis which is consistent with our findings [16]. They discussed that sarcopenia worsens QOL through spinal deformity caused by decreasing muscle mass. Jung et al. also showed that PT is correlated with QOL in osteoporosis patients [18]. Moreover, Miyakoshi et al. concluded that the lower QOL of osteoporosis patients may be associated with deformity of spinal alignment related to generalized muscle weakness [6]. Thus, sarcopenia may worsen QOL through spinal deformity caused by decreasing muscle mass in patients with osteoporosis.

### Pathological mechanisms underlying the correlation between sarcopenia, a low back pain, and reduction of QOL

In this study, sarcopenia affected quality of life and low back pain intensity. A possible explanation is explained by the following pathological mechanisms. It is crucial to maintain an upright position against gravity for humans walking upright on two legs. Anti-gravity muscles such as the multifidus, erector spinae, and vastus lateralis are involved in holding the upright position [19]. These anti-gravity muscles include type l and type II muscle fibers. However, they contain largely type II muscle fibers. Sarcopenia is caused by inflammatory cytokines that cause fatty infiltration of skeletal muscle and induce apoptosis, especially of type II muscle fibers [20]. Fatty infiltration of antigravity muscles such as the multifidus muscle is associated with low back pain [21]. Fat infiltration is also associated with reduced lumbar function [22]. Patients in the sarcopenia group was significantly older than those in the non-sarcopenia group in this study. Age-related type II fiber atrophy and fat infiltration may have affected low back pain and QOL. The ratio of history of vertebral fracture was significantly higher in the sarcopenia group than in the non-sarcopenia group. Dysmobility syndrome is correlating sarcopenia and osteoporosis together with mobility disturbances, obesity, fractures, and falls [23]. Dysmobility syndrome is a useful concept to identify fragility fracture and to do an early intervention, rather than focusing on a single disease, osteoporosis, sarcopenia, or obesity [24]. Dysmobility syndrome is a risk factor of fragility fracture in patients with osteoporosis [25, 26]. Langella et al. investigated the effect of vertebral fractures on spinal deformity. They reported that thoracic kyphosis caused by vertebral body fracture increased the burden on skeletal muscles to maintain alignment in the sagittal plane, resulting in pain [7]. Thus, dysmobility symdrome, combined with sarcopenia and osteoporosis may have caused the fracture to be caused in this study, and the vertebral fracture may have exacerbated the pain. Taken together with our results, the sarcopenia group seems to have exacerbated pain and QOL due to age-related decrease in antigravity muscles and vertebral body fractures resulting from sarcopenia.

### Limitation

This study has several limitations. First, it is a single-center cross-sectional study, there were a small number of cases, and the gender ratio was symmetric. Second, we were unable to assess disease affecting low back pain including lumbar vertebrae herniated disk, ossification of posterior longitudinal ligament, and spinal canal stenosis. These diseases may affect a patient’s low back pain. Third, we showed that sarcopenia exacerbates low back pain and QOL, however, it remains unclear how sarcopenia affects treatments for osteoporosis. It is necessary to perform a multicenter prospective cohort study to investigate the impact of sarcopenia on treatments for osteoporosis.

## Conclusions

In this cross-sectional study, we investigated the effect of sarcopenia on low back pain and QOL in ambulatory patients with osteoporosis. Sarcopenia may worsen low back pain and QOL through spinal deformity caused by decreasing muscle mass in patients with osteoporosis.

## Data Availability

The datasets generated and/or analysed during the current study are available in the University Hospital Medical Information Network Clinical Trial Registry repository, (UMIN000042021). https://center6.umin.ac.jp/cgi-open-bin/ctr_e/ctr_view.cgi?recptno=R000047958.

## Abbreviations

QOL: Quality of life
SMI: Skeletal muscle mass index
JOABPEQ: Japanese Orthopaedic Association Back Pain Evaluation Questionnaire
VAS: Visual Analogue Scale
LL: Lumbar lordosis
PI: Pelvic incidence
SS: Sacral slope
PT: Pelvic tilt
BMI: Body mass index
BMD: Bone mineral density
